# Experimental study for evaluation of a low-cost spray cryotherapy system

**DOI:** 10.31744/einstein_journal/2019AO4533

**Published:** 2019-03-28

**Authors:** Altair da Silva Costa, Andre Miotto, Gustavo Andrade de Paulo, Angelo Paulo Ferrari, Luiz Hirotoshi Ota

**Affiliations:** 1 Hospital Israelita Albert Einstein, São Paulo, SP, Brazil.; 2 Escola Paulista de Medicina, Universidade Federal de São Paulo, São Paulo, SP, Brazil.

**Keywords:** Cryotherapy, Nasal sprays, Bronchoscopy, Swine, Crioterapia, Sprays nasais, Broncoscopia, Suínos

## Abstract

**Objective:**

To evaluate the feasibility and applicability of a low-cost cryotherapy system.

**Methods:**

Experimental study with 25kg Landrace pigs submitted to a longitudinal cervico-thoraco-abdominal incision for exposure of the trachea, thorax and abdomen. The tissues were frozen by continuous spray application at different periods of time (5, 10 and 15 seconds). Spray cryotherapy was performed using a fluorinated gas (tetrafluorethane) delivered at - 47°C temperature (DermaFreeze^®^, Emdutos; ANVISA registration 80409950001; price R$ 394,00). via an adapted, disposable 1.8mm cholangiography catheter (Olympus; price R$ 280,00). The specimens were resected for histopathological analysis.

**Results:**

Thirty samples were obtained from ten different organs and divided according to spray cryotherapy application time. System activation for 5, 10 or 15 seconds led to consumption of 14g, 27g and 40g of gas respectively (average gas consumption, 2.7g/s using a 1.8mm catheter). The system comprising a spray tube and catheter proved user-friendly and effective, with constant gas dispersion and adequate tissue freezing. In spite of effective freezing, microscopy failed to reveal tissue changes. This may have reflected methodological constraints precluding evaluation at tissue damage peak time (48 hours).

**Conclusion:**

The low-cost spray cryotherapy system proved feasible and safe.

## RESUMO

**Objetivo:**

Avaliar a exequibilidade e a aplicação de um sistema de baixo custo de crioterapia.

**Métodos:**

Estudo experimental realizado com um suíno da raça Landrace, 25kg, submetido à cervicotoracolaparotomia longitudinal, com exposição de traqueia, tórax e abdome. Procedemos ao congelamento das estruturas em tempos diferentes (5, 10 e 15 segundos) com jato contínuo. A crioterapia foi realizada com gás fluoretado (tetrafluoretano), na forma de spray em tubo, que atinge a temperatura de -47°C (DermaFreeze^®^, Emdutos; registro ANVISA 80409950001; preço R$ 394,00). A este tubo, adaptamos um cateter descartável de colangiografia de 1,8mm (Olympus; preço R$ 280,00). As peças foram ressecadas para análise histopatológica.

**Resultados:**

Foram obtidas 30 amostras em 10 órgãos diferentes, divididos em três intervalos de tempo distintos. Quando o sistema foi acionado por 5 segundos, gastaram-se 14g de gás; por 10 segundos, 27g; e por 15 segundos, 40g; o gasto médio foi de 2,7g/s pelo cateter de 1,8mm. O sistema confeccionado com tubo de gás e cateter proporcionou resultado efetivo, com dispersão adequada e constante do gás, congelamento adequado e de fácil execução. Apesar da técnica evidenciar congelamento efetivo, na microscopia não houve alteração tecidual. Isso ocorreu porque o pico de lesão tecidual por congelamento ocorre após 48 horas, o que não foi possível avaliar por este método proposto.

**Conclusão:**

O sistema de crioterapia em spray de baixo custo foi exequível e seguro.

## INTRODUCTION

The principles of therapeutic hypothermia have been known and applied since ancient times, and Egyptians and Greeks having been long aware of related anti-inflammatory and anesthetic effects.^(^ ^1^ ^)^

Cryotherapy involves target tissue freezing and thawing. Temperatures ranging from -20°C to -40°C are required to induce the formation of lethal intracellular ice crystals. Working temperatures around -40°C can only be achieved with fast freezing rates (-0.5°C per second) and result in over 90% cell death.^(^ ^1^ ^,^ ^2^ ^)^ Intracellular ice crystals promote cell dehydration and increase of electrolyte concentrations to toxic levels, directing the extracellular fluid to the inside of cells. This effect is also a response to increased cell permeability due to freeze-induced denaturation of cell membrane lipoproteins. Damage to mitochondria and other microorganelles leads to cell death in response to edema and cell rupture, or cryonecrosis.^(^ ^1^ ^-^ ^3^ ^)^

Tissues such as skin, mucous membranes and granulation tissue are highly sensitive to freeze-induced destruction, whereas other tissues, like fat, fibrosis, cartilage and connective tissues are less sensitive (cryosensitive and cryoresistant tissues, respectively).^(^ ^1^ ^-^ ^3^ ^)^

The development of novel technologies and materials allowed more effective and faster tissue freezing. Cryotherapy enjoyed particular growth in dermatology, as skin lesions are exposed and easy to access. Cryotherapy is currently a well-established dermatological treatment modality, with potential application in other areas.^(^ ^4^ ^,^ ^5^ ^)^

Airway cryotherapy is applied by means of sophisticated devices and cooling agents such as nitrogen spray or catheter-based delivery of carbon dioxide, while fluorinated gases are not used.^(^ ^3^ ^,^ ^5^ ^)^

## OBJECTIVE

To evaluate the feasibility and practical application of a low-cost cryotherapy system.

## METHODS

Experimental study with Landrace pigs weighing 20 to 25kg. Animals were cared for according to the Guide for the Care and Use of Laboratory Animal standards (Institute of Laboratory Animal Resources; National Academy of Sciences, Washington D.C., 1996) and to Ethical Principles in Animal Experimentation, provided by Brazilian laws.^(^ ^6^ ^-^ ^8^ ^)^ Animals used in this study had been submitted to previous procedures as part of an advanced therapeutic digestive endoscopy workshop taking place on the same day.

**Figure 1:**
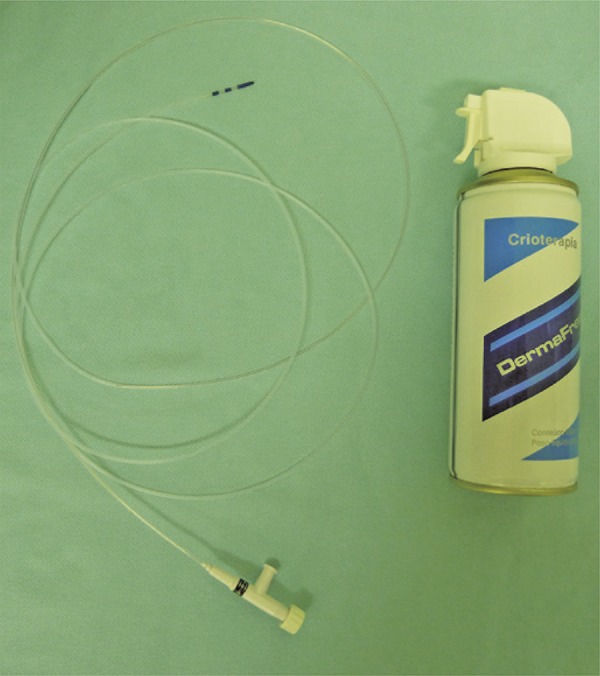
Fluorinated gas tube (DermaFreeze®) and disposable endoscopic cholangiography catheter (PR-225Q/PR427G, 1.8mm, Olympus®)

Cryotherapy was performed using a disposable 1.8-mm cholangiography catheter (Olympus; R$ 280,00) ( Figure 1 ) connected to a spray tube containing fluorinated gas (tetrafluorethane), delivered at -47°C temperature (DermaFreeze^®^, Emdutos, ANVISA [Brazilian Health Regulatory Agency] registration 80409950001; R$ 394,00).^(^ ^1^ ^)^

Animals were placed on operating tables on supine position, and submitted to longitudinal cervicothoracoabdominal incision, to expose the trachea, thorax and abdomen. Intact tissue samples were collected prior to cryotherapy for comparative analysis. Tissues were submitted to cryotherapy via continuous application of fluorinated gas for 5, 10 or 15 seconds. Specimens were resected for histopathological analysis within approximately 15 minutes of freezing. Animals were kept anesthetized, tubed and mechanically ventilated until euthanasia.

**Figure 2:**
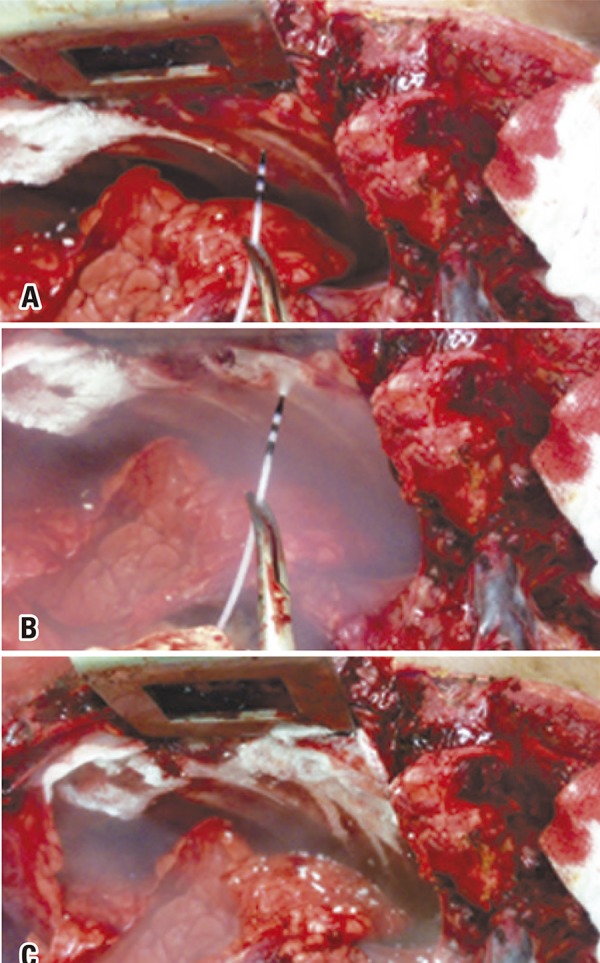
Freezing technique applied to the pleura. (A) Catheter preparation and positioning. (B) Fluorinated gas spray application. (C) Immediate freezing results

Cold-induced lesions were produced in the following regions: larynx, trachea, esophagus, lung, parietal pleura ( Figure 2 ), spleen, liver, stomach, small intestine and heart-atrium. Specimens were immersed in 10% neutral, buffered formaldehyde solution for 24 hours.

Representative fragments (average thickness, 5mm) were obtained from all tissues. Samples were dehydrated and embedded in paraffin. Histological sections (5μm thick) were stained with hematoxylin/eosin (HE) and analyzed by optical microscopy under 40x and 100x magnification.

This study was approved by the Ethics Committee for the Use of Animals of the participating institutions (CEUA/UNIFESP 2402080414 and CEUA-HIAE 1919-13.

## RESULTS

Thirty samples of 10 different organs were collected following fluorinated gas spray application for different periods of time. System activation for 5, 10 or 15 seconds led to consumption of 14g, 27g and 40g of gas respectively (average gas consumption, 2.7g/s, delivered via a 1.8mm catheter).

**Figure 3:**
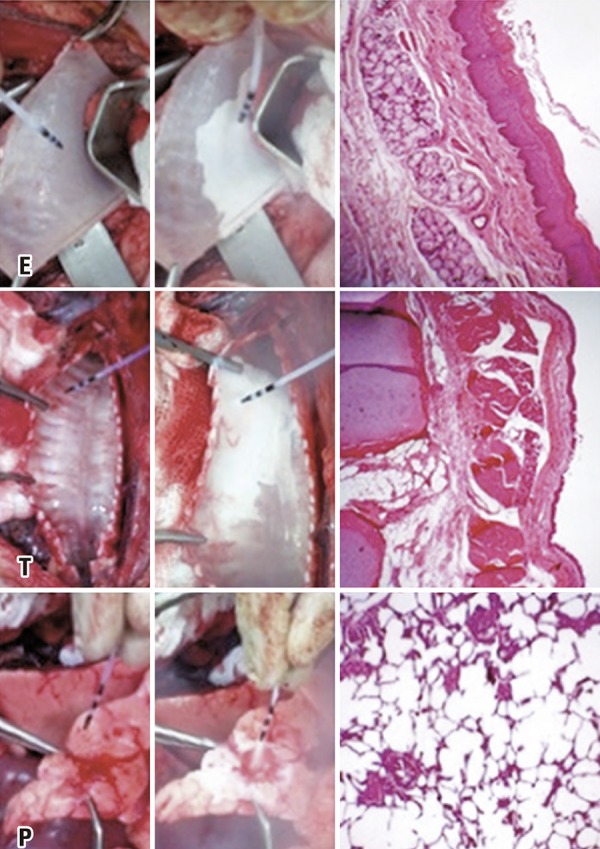
Freezing and histology of esophagus (E), trachea (T) and lung (P) specimens

Freezing of target structures became evident following spray application, as shown in figures 2 and 3 . The system employed ( *i.e* ., spray tube and 1.8mm catheter) was not associated with complications.

**Figure 4:**
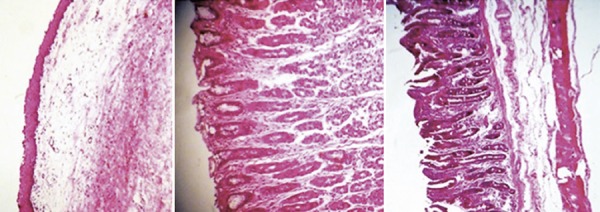
Histology of thawed larynx, stomach and small intestine tissue samples

**Figure 5:**
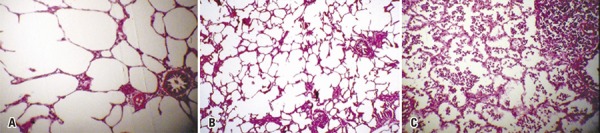
Histological sections of the lung. (A) Not exposed to the gas. (B) Exposure time of 10 seconds. (C) Exposure time of 15 seconds

In this study, tissue resection was performed within 15 minutes of freezing; this time period proved sufficient for thawing to occur. Tissue samples were kept in formaldehyde. Histological analysis of HE-stained specimens failed to reveal differences between control and cryotherapy-treated samples, regardless of freezing time ( Figures 3 , 4 and 5 ).

## DISCUSSION

In thermodynamics, the Joule-Thomson effect describes temperature changes in pressurized gases or fluids passing through a valve provided they are contained in a closed, isolated vial protected against heat exchange with the environment. With the exception of hydrogen, helium and neon, expanding gases cool down at room temperature – the Joule-Thomson experiment.^(^ ^1^ ^,^ ^2^ ^,^ ^9^ ^)^

Gases liquefied under pressure can be stored in spray tubes or cans. Spray deodorants for daily use are one example of this procedure. As the gas is released, cooling of the gas itself and of the area around the valve can be easily perceived. Based on this physical principle, certain gases stored in closed vials can be used for therapeutic cryotherapy. One of such gases is the fluorinated gas tetrafluoroethane, or DermaFreeze^®^.^(^ ^1^ ^,^ ^2^ ^,^ ^5^ ^)^

In cryotherapy, low temperatures are applied to induce cell changes and vasoconstriction, reducing inflammation and promoting analgesia or even tissue necrosis^(^ ^1^ ^,^ ^5^ ^)^ according to cold exposure intensity and time. The amount of tissue destroyed by cryotherapy depends on several factors, such as freezing and thawing rates, minimum temperatures achieved, cell water content, number of freezing-thawing cycles and tissue sensitivity.^(^ ^9^ ^-^ ^11^ ^)^

Cryotherapy is known for its primary application in skin, mucous membranes and the airways. However, the type of system or gas employed is a limiting factor. Liquid nitrogen must comply with strict safety standards, involves storing logistics, requires specific storage vials and must be handled by trained personnel. Liquid nitrogen may be used in clinics and outpatient services;^(^ ^2^ ^,^ ^6^ ^)^ the spray form is required for airway applications. The spray cryotherapy device TrueFreeze System (CSA Medical) consists of liquid nitrogen delivery via a flexible catheter passed through the working channel of a bronchoscope.^(^ ^3^ ^,^ ^5^ ^,^ ^10^ ^,^ ^12^ ^)^ However, it is a large piece of equipment unavailable in Brazil and therefore associated with cost and access constraints.

Airway cryotherapy use is limited in Brazil given its high costs and other requirements, such as imports, registration by the National Health Surveillance Agency *(* ANVISA *- Agência Nacional de Vigilância Sanitária)* and tax burden. The development of novel, national technologies with potential airway applicability stemmed from these constraints.

This initial experimental study aimed to test the feasibility of the system in question. The system comprising a gas tube and endoscopy catheter proved user-friendly and effective, with proper constant gas dispersion and adequate tissue freezing.

Spray cryotherapy with nitrogen or catheter-based carbon dioxide delivery are occasionally used in bronchoscopy.^(^ ^5^ ^)^ Sadly, airway application of spray cryotherapy with fluorinated gas is still limited by lack of investigation.

In spite of effective freezing from a macroscopic perspective, the technique investigated in this study failed to induce microscopic tissue changes. This may have reflected the short time of frozen tissue permanence in the body. Animals in this trial were destined to euthanasia and surgical specimens were resected within 15 minutes of freezing. Cryotherapy-induced tissue damage peaks within 48 hours of freezing; therefore, this effect could not be evaluated due to methodological constraints.^(^ ^1^ ^,^ ^5^ ^,^ ^11^ ^,^ ^12^ ^)^

Further studies are warranted to determine the extension of damage induced by fluorinated gas-based respiratory system cryotherapy.

## CONCLUSION

The low-cost spray cryotherapy system tested in this experimental study proved feasible, practical and safe.
